# Successful correction of hemophilia by CRISPR/Cas9 genome editing *in vivo*: delivery vector and immune responses are the key to success

**DOI:** 10.15252/emmm.201606325

**Published:** 2016-04-04

**Authors:** Tuan Huy Nguyen, Ignacio Anegon

**Affiliations:** ^1^ INSERMUMR 1064‐Center for Research in Transplantation and ImmunologyNantesFrance; ^2^ ITUNCHU NantesNantesFrance; ^3^ Faculté de MédecineUniversité de NantesNantesFrance

**Keywords:** Genetics, Gene Therapy & Genetic Disease, Haematology

## Abstract

Hemophilia B is a serious hemostasis disorder due to mutations of the factor IX gene in the X chromosome. Gene therapy has gained momentum in recent years as a therapeutic option for hemophilia B. In hemophilia, reconstitution with a mere 1–2% of the clotting factor improves the quality of life, while 5–20% suffices to ameliorate the bleeding disorder. A paper by Guan *et al* (2016) in this issue of *EMBO Molecular Medicine* reports on the direct CRISPRs/Cas9‐mediated correction in the liver of a hemophilia‐causing point mutation in FIX.

The main therapeutic goal, as with other monogenic disorders, is to restore gene function permanently, while limiting cell toxicity, genome alterations or induction of harmful immune responses against the components of the gene therapy system used and against the products of the transgene.

To avoid genotoxicity and achieve physiological levels of transgene expression, gene‐specific genome editing or site‐specific gene addition is preferable to the random integration of expression cassettes, which could result in insertional mutagenesis and cancer as well as silencing of transgene expression.

Gene therapy without genome editing using recombinant adeno‐associated virus (rAAV) containing an expression cassette has been tested in clinical gene therapy trials to treat hemophilia B with excellent results at 5‐year post‐treatment follow‐up (Nathwani *et al*, 2014). As rAAV largely persists in an episomal state, the long‐lasting therapeutic effects observed in the clinical trial are probably due to the fact that recruited patients are adults and thus the liver is quiescent and consequently hepatocyte renewal is very slow. The pre‐existence or the appearance of anti‐AAV cellular immunity is an obstacle to the persistence of genetically corrected cells (Manno *et al*, 2006).

Repair of the defective gene or gene replacement by targeted integration into the genome would allow long‐term expression of the introduced sequence, but this would depend on the type of delivery method used. Gene‐specific nucleases such as ZFNs, TALENs, or CRISPR/Cas9 upon DNA cleavage of the targeted sequence allow a several fold increase in the insertion into the cleavage point of DNA repairing sequences that are delivered simultaneously to the nucleases.

In this respect, rAAV were used for hepatic delivery in adult and newborn mice of ZFNs to intron 1 of the factor 9 gene (*F9*) and as DNA donor an acceptor splicing sequence and the cDNA of exons 2–8 to obtain production of factor IX in a humanized murine model of hemophilia B (Li *et al*, 2011).

More recently, rAAV were used for hepatic delivery in hemophilia B mice of a promoter‐less F9 cDNA preceded by a 2A‐peptide coding sequence flanked by homology arms to integrate the repairing DNA just upstream of the stop codon of albumin, a highly expressed gene in the liver. The target genomic sequence was not cleaved by nucleases (nuclease‐free approach), and the F9 cDNA was integrated just by means of spontaneous homologous recombination possibly favored by the rAAV flanking sequences (Barzel *et al*, 2015). On‐target in the albumin locus was achieved at a level of up to 0.5% and restored 7–20% of normal FIX expression. This approach thus avoids potential off‐effects of nucleases.

A recent report involving instead site‐specific insertion in the liver described the correction of a mutation causing fumarylacetoacetate hydrolase (FAH) deficiency, which at variance with hemophilia, confers a selective advantage to hepatocytes in which correction has taken place. In this paper, genome editing was performed using CRISPR/Cas and ssODNs that directly introduced the appropriate sequence into the defective gene (Yin *et al*, 2014).

As mentioned above, the paper by Guan *et al* (2016) in this issue of *EMBO Molecular Medicine* reports on the direct CRISPRs/Cas9‐based correction in the liver of a hemophilia‐causing point mutation using an approach similar to the one used to correct FAH deficiency but without a selective advantage for corrected hepatocytes. The authors first describe a male hemophilia B patient with a novel *F9* missense mutation [g.31094T>G (p.Tyr371Asp)]. The authors then elegantly demonstrate that this mutation is indeed responsible for hemophilia by generating a mouse strain containing the mutation and presenting with normal levels of F9 but with the hemostasis defect. Finally, the authors correct the mouse mutation *in vivo* by hydrodynamic tail injection of a plasmid encoding Cas9 and the sgRNA along with either ssODN (120 nt) or long donor DNA (with homology arms of 0.4 kb) containing the corrected sequence. Using both DNA donors, hemostasis was significantly corrected with up to 0.56 and 1.5% of hepatocytes displaying a corrected genotype using ssODNs and the long DNA, respectively. A higher proportion of hepatocytes showed indels without correction indicating DNA cleavage with non‐homologous end joining repair and without insertion of the donor DNA. This treatment did not induce hepatocyte damage. The authors then moved to the simultaneous use of two adenoviral vectors, one encoding Cas9 and the second one the sgRNA and the long DNA donor. Despite obtaining a high frequency of hepatocyte correction (5.5%) at early time points, no correction of hemostasis was observed at later time points. This lack of biological response despite genomic correction was associated with high inflammation in the liver and hepatocyte death. This is most likely due to an anti‐adenoviral immune response that destroyed the genome‐edited hepatocytes, although this is not specifically reported.

It would appear therefore that the delivery of the genetic components of the CRISPR/Cas9 system and DNA donor albeit not necessarily very efficient (~1% of corrected hepatocytes) needs to be such that inflammation and immune responses directed against any of the gene repair components are avoided. The adenoviral vectors used in the study induced inflammation and likely immune responses but helper‐dependent adenoviral vectors or rAAV are less immunogenic. Despite this, rAAV have the best efficacy/safety profile of any viral vector when considering applications to genetic diseases and it would be important in the future to evaluate the efficiency of rAAV to deliver the different components of the CRISPR/Cas system as well as the donor repairing DNA.

Hydrodynamic injection is not considered to be clinically feasible at the present time, despite that it has been performed with a DNA vector in liver segments of pigs and humans (Khorsandi *et al*, 2008) and with AAV vectors in the legs of non‐human primates (Toromanoff *et al*, 2008).

Some technical aspects of the study by Guan *et al* (2016) may be improved upon in the future with the aim to increase the efficacy of genome editing. Firstly, modified ssODNs have shown superior genome editing efficacy (Renaud *et al*, 2016), possibly by protecting the ssODNs from degradation, and thus would be an interesting future alternative to increase genome editing efficacy. Secondly, since the efficacy of insertion of DNA repair molecules increases when using different forms of Cas9 molecule, protein>mRNA>DNA (Ménoret *et al*, 2015), new *in vivo* delivery methods of Cas9 as a protein might increase the efficacy of the procedure.

The paper by Guan *et al* (2016) analyzed off‐target effects by *in silico* definition of potential targets followed by the T7 assay. It has been shown that more and new off targets can be identified when other methods are used (Gabriel *et al*, 2011) and T7 analysis is not very sensitive when compared to techniques such as NGS analysis. Therefore, potential off‐target effects need to be better defined before going into clinical trials.

Previous reports have shown that ZFNs (Li *et al*, 2011) and even a nuclease‐free approach (Barzel *et al*, 2015) are also efficient and since they insert *F9* cDNA under the control of the albumin or *F9* promoters, they have potential application in any case of hemophilia B regardless of the mutation. The genome editing strategy using CRISPR/Cas and ssODNs described in the manuscript by Guan *et al* (2016) would have to be tailored to each mutation and therefore depends on the generation of different sgRNAs that may have different efficacies.

All the above‐mentioned strategies have the clinical potential to also treat hemophilia A, which is more common than hemophilia B, as well as several liver genetic diseases. The paper by Guan *et al* (2016) is therefore important and encouraging and illustrates how the gene delivery vehicle and the role of immune responses are of key importance in the efficacy, biosafety, and duration of successful therapeutic genome editing.
